# Transforming of Triptolide into Characteristic Metabolites by the Gut Microbiota

**DOI:** 10.3390/molecules25030606

**Published:** 2020-01-30

**Authors:** Ran Peng, Shu-Rong Ma, Jie Fu, Pei Han, Li-Bin Pan, Zheng-Wei Zhang, Hang Yu, Yan Wang

**Affiliations:** State Key Laboratory of Bioactive Substance and Function of Natural Medicines, Institute of Materia Medica, Chinese Academy of Medical Sciences and Peking Union Medical College, Beijing 100050, China; pengran@imm.ac.cn (R.P.); mashurong@imm.ac.cn (S.-R.M.); fujie@imm.ac.cn (J.F.); hanpei@imm.ac.cn (P.H.);

**Keywords:** triptolide, gut microbiota, liver microsome, metabolites, LC-MS/MS, LC/MS^n^-IT-TOF

## Abstract

The importance of the gut microbiota in drug metabolism, especially in that of nonabsorbable drugs, has become known. The aim of this study was to explore the metabolites of triptolide by the gut microbiota. With high-performance liquid chromatography coupled with tandem mass spectrometry and ion trap time-of-flight multistage mass spectrometry (LC-MS/MS and LC/MS^n^-IT-TOF), four metabolites of triptolide (M1, M2, M3, and M4) were found in the intestinal contents of rats. M1 and M2, were isomeric monocarbonyl-hydroxyl-substituted metabolites with molecular weights of 390. M3 and M4 were isomeric dehydrogenated metabolites with molecular weights of 356. Among the four metabolites, the dehydrogenated metabolites (M3 and M4) were reported in the gut microbiota for the first time. The metabolic behaviors of triptolide in the gut microbiota and liver microsomes of rats were further compared. The monocarbonyl-hydroxyl-substituted metabolites (M1 and M2) were generated in both systems, and another monohydroxylated metabolite (M5) was found only in the liver microsomes. The combined results suggested that the metabolism of triptolide in the gut microbiota was specific, with two characteristic, dehydrogenated metabolites. This investigation might provide a theoretical basis for the elucidation of the metabolism mechanism of triptolide and guide its proper application in clinical administration.

## 1. Introduction

Triptolide is an epoxy diterpene lactone extracted from the dried roots of the celastraceae plant, which is one of the main active ingredients of *Tripterygium wilfordii* Hook F. It has a number of pharmacological activities, including immunomodulation, anti-inflammatory, and antitumor activities. Hence, extracts of *T. wilfordii* have been used to treat autoimmune diseases and inflammation [1]. Triptolide, the main active ingredient of *T. wilfordii* [2], is a cytotoxic agent and chemotherapeutic agent, which can not only inhibit tumor growth and metastasis, but also directly induce tumor cell death [3]. However, with the wide use of triptolide, many studies and clinical case reports have shown that it has toxic effects on organs such as the liver, kidney, spleen, gastrointestinal tract, and heart [4]. One of its most severe adverse effects observed in the clinical use is hepatotoxicity, Zhao et al. applied an LC/MS-based metabolomic analysis to characterize the metabolomic change in serum and liver induced by triptolide in mice [5]. The clinical application of triptolide is limited by its serious side effects and its further development is thus restricted. At present, some metabolites of triptolide and related derivatives have been reported [6]. Some researchers found that the major metabolic pathways of triptolide in rats were hydroxylation, sulphation, and glutathione (GSH) conjugation [7]. In terms of the metabolic pattern of triptolide *in vivo*, there are four commonly reported metabolites with molecular weights of 376, 376, 374, and 390. Two of them are monohydroxylated metabolites, and one is carbonylated metabolite. The last one with an *m/z* of 390, is a hydroxy and carbonylated metabolite [8].

In recent years, researchers have conducted a systematic study of drug metabolism by gut microbiota. After administration, the drug usually undergoes chemical modification, producing metabolites that can have different functional and toxicological properties compared to those of the precursor drug [9]. Most drugs are delivered orally, and symbiotic microorganisms can be encountered in the small and large intestines. These microorganisms collectively encode 150 times more genes than the human genome, which contains a potential enzyme library for drug metabolism. Examples of interactions between the gut microbiome and drugs or drug metabolites have previously been reported, suggesting the systematic pharmacological effect of these microbes on orally administered drugs. The modification function of intestinal microorganisms can lead to the activation of these compounds (such as sulfasalazine [10]), inactivation (such as digoxin [11]), or poisoning (such as irinotecan [12]). Notably, some drugs can only be transformed by specific microorganisms [11,13,14], but these are rare cases. Peng et al. showed that triptolide could be metabolized *in vitro* by hydroxylation reaction and found a metabolite (*m/z* 377) in the intestinal incubations of Wister rats [15]. The diversity and complexity of gut microbiota composition and function makes microbiome-drug metabolism a complicated process and needs further exploration. The advent of “-omics” techniques allows for us to investigate the relationship between microbiome and drugs. Our study focused on the metabolism of triptolide in gut microbiota. In this research, the metabolism of triptolide in the gut microbiota of Sprague–Dawley (SD) rats was studied *in vitro,* and possible mass cleavage pathways were proposed using LC-MS/MS and LC/MS^n^-IT-TOF. The triptolide metabolites from liver microsomes of SD rats were compared with those from the gut microbiota. Among them, two new metabolites in the gut microbiota with a molecular weight of 356 were discovered for the first time. The study of the role of intestinal microorganisms on the metabolism of triptolide might help to explain the mechanisms of its detoxification and offer new ideas for the pharmacological and toxicological mechanisms of triptolide, and it is expected to further provide guidance for its clinical application.

## 2. Results

### 2.1. Participation of Gut Microbes in the Metabolism of Triptolide

**Figure 1:**
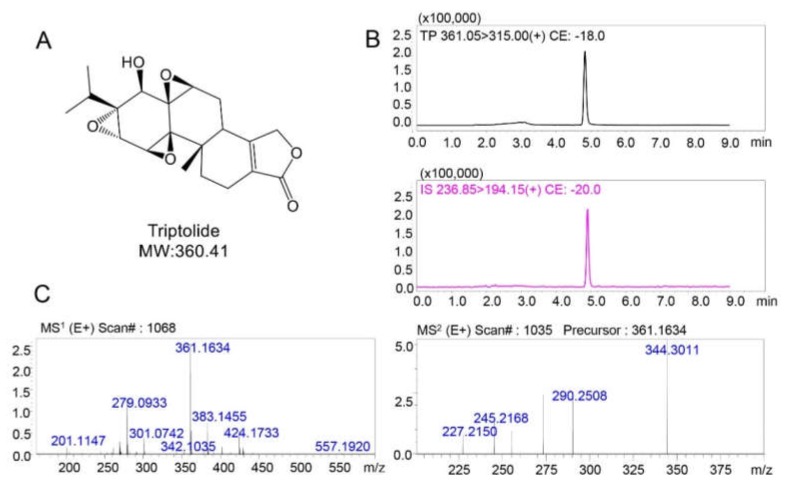
The structure and mass spectra of triptolide. (**A**) The structural formula of triptolide. (**B**) Extracted ion chromatogram (EIC) spectra of triptolide and the internal standard (internal standard (IS), carbamazepine). (**C**) The MS^1^ and MS^2^ mass spectra of triptolide acquired by LC/MS^n^-IT-TOF.

The aim of the study was to elucidate the unique metabolic profile of triptolide in the gut microbiota and to compare the differences in metabolic pathways between the gut and liver. The molecular structure of triptolide is shown in Figure 1A. LC-MS/MS was used to determine the level of triptolide in the samples and validation results are provided in Supplementary Data Table S1. The retention times of triptolide and the internal standard (carbamazepine) were 4.2 min and 4.7 min, respectively, as shown in Figure 1B. Furthermore, LC/MS^n^-IT-TOF was used to identify the ion fragments of triptolide, and the MS^1^ and MS^2^ spectrum of triptolide is shown in Figure 1C. Triptolide exhibited ions at *m/z* = 361, which was the [M + H]^+^ peak, and *m/z* = 383, which was the [M + Na]^+^ peak, and the main MS^2^ fragments were at (*m/z*) 344, 290, 245, and 227.

**Figure 2:**
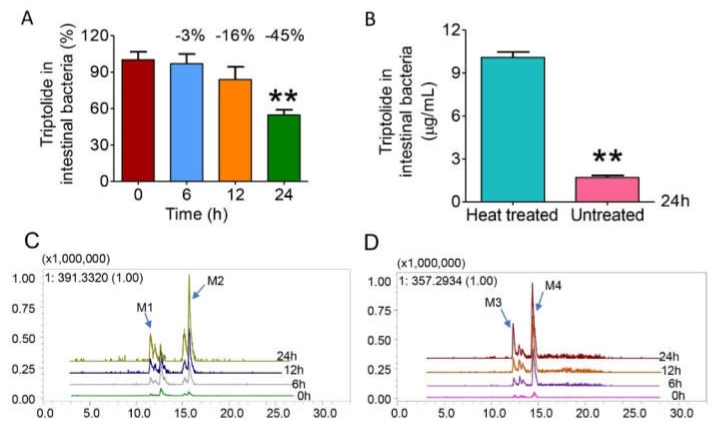
Triptolide could be metabolized in the gut microbiota. (**A**) The level of triptolide decreased during incubation with rat intestinal bacteria after 0 h, 6 h, 12 h, and 24 h. (**B**) Intestinal bacteria mainly participated in the metabolism of triptolide by comparison between untreated and heat-inactivated intestinal contents. (**C**) The extracted ion chromatograms (EICs) showed that the levels of the possible metabolites M1 and M2 increased with increasing time. (**D**) The EICs showed that the levels of possible metabolites M3 and M4 increased with increasing time. Data are presented as mean ± SD, and two-tailed Student’s t test were used for analysis (** *p* < 0.01).

To explore whether the gut microbiota was involved in the metabolism of triptolide, the colon contents of six SD rats were incubated with triptolide, and heat- treated inactivated colon contents were incubated as a negative control. The reaction was stopped after coincubation for 0 h, 6 h, 12 h, and 24 h. The level of triptolide in the culture was detected by LC-MS/MS, which is shown in Figure 2A. The quantity of triptolide in the system showed a gradual decreasing trend over time, with a drop of 3% at 6 h, 16% at 12 h, and 45% at 24 h (*p* < 0.01). This result indicates that the gut microbiota of rats might have a metabolic effect on triptolide. In contrast, the heat-inactivated gut microbiota hardly metabolized triptolide (shown in Figure 2B), suggesting that the observed decline of triptolide in Figure 2A was the result of co-metabolism by living microbiota, which certified the role of the gut microbiota in the metabolism of triptolide.

### 2.2. Metabolism of Triptolide in the Gut Microbiota

**Figure 3:**
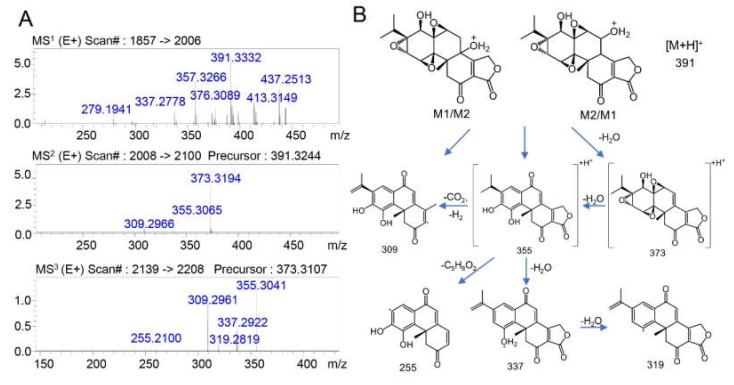
(**A**) The MS^n^ data of the triptolide metabolites M1 and M2. (**B**) Possible structures and mass spectrometric cleavage pathway of metabolites M1 and M2.

**Figure 4:**
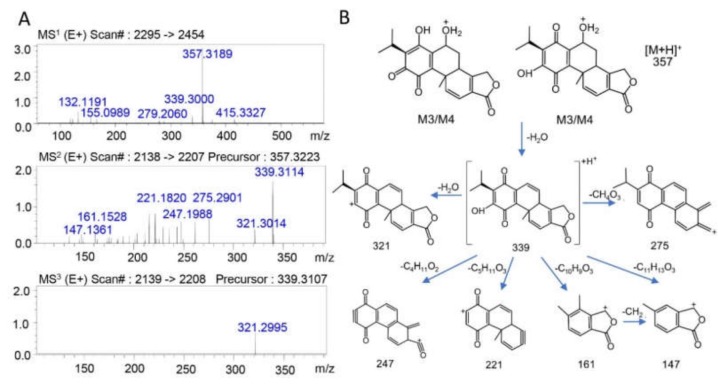
(**A**) The MS^n^ data of the triptolide metabolites M3 and M4. (**B**) Possible structures and mass spectrometric cleavage pathway of the metabolites M3 and M4.

To explore the metabolites of triptolide in the gut microbiota, LC/MS^n^-IT-TOF was used to search and deduce the structures of the metabolites in the reaction system. Among them, 4 metabolites (M1–M4) were found, as shown in Figure 2C,D, with retention times of 11.5 min, 15.7 min, 12.4 min, and 14.8 min, respectively (on LC/MS^n^-IT-TOF). The abundance of each metabolite increased significantly over time (in Supplementary Data Table S2), but they were not detected in the inactivated gut microbiota system, which indicated that M1–M4 might be the metabolites of triptolide cometabolized by gut microbiota, further elucidating their possible structures (Figure 3 and Figure 4).

The possible structures of M1 and M2 and the spectra of the MS^n^ fragments are shown in Figure 3A,B. Both M1 and M2 exhibited an ion of *m/z* = 391 as their [M + H]^+^ ions, and the retention times were 11.5 min and 15.7 min, respectively. According to the fragmentation of triptolide, the structures of the generated fragments were proposed as follows: the ions of *m/z* 373 and 355 were produced with a loss of H_2_O (−18, *m/z* 373) or 2 H_2_O (−36, *m/z* 355). Then, fragments with a further loss of H_2_O (−18, *m/z* 337), 2 H_2_O (−18, *m/z* 319), CO_2_H_2_ (−46, *m/z* 309), or C_5_H_8_O_2_ (−100, *m/z* 255) were possibly generated, as shown in Figure 3A. Based on the possible metabolic pathways, molecular weights, and mass spectral cleavage pathways, M1 and M2 were proposed to be monohydroxy-carbonylated metabolites of triptolide, which was consistent with the reported metabolite in urine [8,16].

Apart from this, two additional metabolites (M3, M4) were observed with the smaller molecular weight of 356. The possible structures of M3 and M4 and the spectra of MS^n^ fragments are shown in Figure 4A,B. The *m/z* of the [M + H]^+^ ions of both M3 and M4 was 357, and the retention times were 12.4 min and 14.8 min, respectively, as shown in Figure 4A. The proposed cleavage pathways were as follows: First, M3 or M4 could have lost a molecule of H_2_O and produced the ion of *m/z* = 339 (−18). Then, fragments with a further loss of H_2_O (−18, *m/z* 321), CH_4_O_3_ (−64, *m/z* 275), C_4_H_12_O_2_ (−92, *m/z* 247), C_5_H_10_O_3_ (−118, *m/z* 221), C_10_H_10_O_3_ (−178, *m/z* 161), or C_11_H_12_O_3_ (−192, *m/z* 147) could have been generated, and the possible structures are shown in Figure 4A. Based on the possible metabolic pathways, molecular weights, and mass spectral fragmentations, M3 and M4 were tentatively identified as dehydrogenated metabolites of triptolide, and the structures of these two metabolites have been reported for the first time.

### 2.3. Metabolism of Triptolide in Liver Microsomes

**Figure 5:**
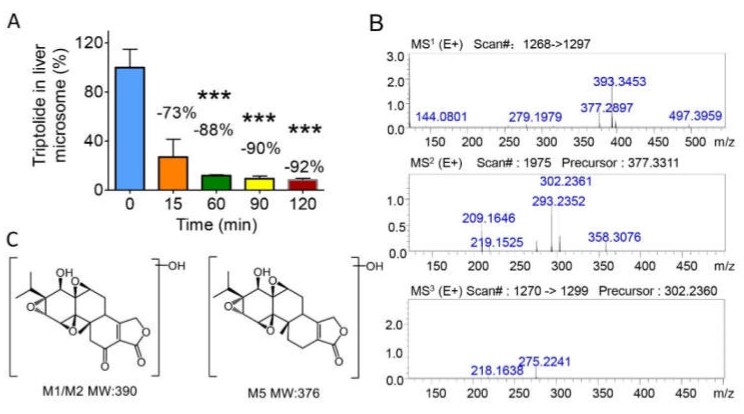
The metabolites of triptolide in liver microsomes. (**A**) The level of triptolide incubated with rat liver microsomes at different time points (0 min, 15 min, 60 min, 90 min, and 120 min). (**B**) The MS^n^ data of the triptolide metabolite M5 (molecular weight: 376) in liver microsomes. (**C**) The possible structure of the triptolide metabolites M1/M2 and M5 in rat liver microsomes. Data are presented as mean ± SD, and two-tailed Student’s t test were used for analysis (*** *p* < 0.001).

To compare the differences in the metabolic pathways of triptolide between liver microsomes and the gut microbiota, microsomes of SD rats were prepared and then incubated with triptolide. In the microsome system, after incubation with triptolide for 0 min, 15 min, 60 min, 90 min, and 120 min, the reaction was stopped with cold acetonitrile, and samples were prepared for LC-MS/MS analysis. The level of triptolide changed over time, and the results are shown in Figure 5A. With increasing incubation time, the triptolide content in the microsomal incubation system showed a significant decrease after 2 h, during which the triptolide level was reduced by 73% in 15 min, 88% in 60 min (*P* < 0.001) and 92% in 120 min (*P* < 0.001). These results indicated that triptolide could also be metabolized by the liver.

At the same time, we found three metabolites (M1, M2, and M5) in the microsome culture system. The possible structures of M1, M2 and M5 are shown in Figure 5C. All fragment ions of M1 and M2 were the same as those found in the gut microbiota, suggesting that M1 and M2 could be common metabolites in the liver and gut microbiota, as summarized in Table 1. The [M + H]^+^ ions of M1 and M2 were *m/z* = 391, and the retention times for M1 and M2 were 11.5 min and 15.7 min, respectively. Additionally, a possible metabolite with a molecular weight of 376 was found in the liver microsomes, namely, M5. The retention time of M5 was 13.1 min. Figure 5B shows the MS^n^ mass spectra of M5 obtained by LC/MS^n^-IT-TOF. According to the fragment ions and known *in vivo* metabolites [6], the structure of M5 was proposed and is shown in Figure 5C.

## 3. Discussion

Although triptolide (TP) is effective in treating various inflammatory and autoimmune diseases, its clinical application is limited due to its toxicity [17,18]. Apart from the toxicity of triptolide itself, the possible toxic metabolites may work together, hence, it is important to understand its metabolic characteristics. Currently, the marketed drugs containing triptolide are mainly oral drugs. Inevitably, triptolide may also interact with the intestinal flora. From the perspective of intestinal bacteria, this article revealed that the gut microbiota participated in the metabolism of triptolide. A total of 4 main metabolites were found, two of which were monocarbonyl-hydroxyl-substituted metabolites and two of which were dehydrogenated metabolites with molecular weights of 390, 390, and 356, 356, respectively. Among them, the dehydrogenated metabolites had not been previously reported in the literature. These are the newly discovered isomeric metabolites, their structures were initially identified based on ion trap-time of flight multistage mass spectrometry, and their cleavage pathways were analyzed. The discovered new metabolites might present different characters of toxicity for that several functional groups (which were associated with toxicities) have been changed after transformation, which needs further investigation. In the liver microsomal reaction system, the metabolites of triptolide were two monocarbonyl monohydroxy-substituted metabolites (M1 and M2, with molecular weights of 390 and 390) and one monohydroxylated metabolite (M5, with a molecular weight of 376). According to the literature, metabolites with a molecular weight of 376 might be mainly produced via cytochrome P450 (CYP3A) [19]. Peng et al. [15] have reported a hydroxylated metabolite of *m/z* 377, but we found that similar peak area level of *m/z* 377 was presented in both untreated and heat-treated bacterial incubation. The peak area of this compound did not increase with time. Hence, we predicted that this metabolite (*m/z* = 377) might not be mainly produced by the intestinal bacterial incubation in this investigation. The reasons of differences might be involved in the different composition of gut microbiota in SD rats and Wister rats and the different formulation of anaerobic medium for incubation. These results indicated that the intestinal bacteria may have a characteristic metabolic pattern for triptolide that is different from that in the liver.

While further research in depth still needed, because of some limitations including: the selection of animal models, and the common limitations existed in the *in vitro* bacterial system (not all the intestinal bacteria could be cultured in the medium).

Triptolide has been reported to have a variety of significant pharmacological activities, but its toxicity and the associated side effects cannot be ignored. At present, its pharmacological and toxicological mechanisms are unclear. The study of the metabolism of triptolide by intestinal flora might provide a material basis for its medicinal effects and toxicity. With further research on the biological activity of intestinal flora-derived metabolites, the pharmacodynamic mechanism of triptolide might be explored, which is helpful to provide new ideas for the reduction of its toxicity and guide its clinical application.

## 4. Materials and Methods

### 4.1. Instruments and Reagents

Triptolide and carbamazepine were purchased from Solarbio Life Sciences Co., Ltd. (Beijing, China). The purity of the compounds was higher than 98% (HPLC). HPLC-grade acetonitrile, and methanol were purchased from Fisher Scientific (Fair Lawn, NJ, USA). An HPLC-MS/MS 8050 system from Shimadzu Corporation (Kyoto, Japan) was employed for the quantitative determination of triptolide and HPLC-electrospray ionization-ion trap-time of fight mass spectrometry (Shimadzu, Kyoto, Japan) was used to identify the structure of metabolites of triptolide in intestinal bacteria and liver microsomes. A WH-681 vortex mixer was purchased from Jintan Shenglan Instrument Manufacturing Co., Ltd., and a small-scale refrigerated high-speed centrifuge was purchased from Eppendorf (Hamburg, Germany).

### 4.2. Animals

SD rats (180–200 g, male) were provided by the Institute of Experimental Animals, Chinese Academy of Medical Sciences (Beijing, China). These animals had free access to food and water and were housed with, a 12 h light/dark cycle at 22–24 °C and 40%–60% relative humidity. The feed for animals was obtained from Beijing Keao Xieli Feed Co., Ltd., which contains: corn, fish meal, flour, bran, sodium chloride, calcium hydrogen phosphate, stone powder, vitamins and trace elements, amino acids, etc. This study was conducted with the permission and guidance of the Experimental Animal Ethics Committee of the Chinese Academy of Medical Sciences and the Peking Union Medical College. All steps refer to the “Organizational Guidelines and Ethics Guidelines of the Experimental Animal Ethics Committee”.

### 4.3. Determination of Triptolide by LC-MS/MS

The quantitative determination of triptolide was performed by LC-MS/MS 8050 equipped with an ESI ionization source. The analytes were separated through an Alltima C_18_ column (100 mm × 2.1 mm, 5 μm, Grace, England). The temperature of the column oven was 30 °C, and the flow rate was 0.6 mL/min. The mobile phase consisted of formic acid: water (0.1:100, *v*/*v*) (as mobile phase A) and acetonitrile (as mobile phase B). The elution gradient conditions (A:B) were as follows: 0.01 min, 50:50; 1.00 min, 50:50; 5.00 min, 10:90; 7.00 min, 5:95; 7.01 min, 50:50; and 10 min, stop. The detection was carried out in positive MRM mode, with mass transitions for triptolide and the internal standard (IS, carbamazepine) of 361.05 → 343.00 (Q1 Pre Bias: −12 V, CE: −18.0 V, Q3 Pre Bias: −14.0 V), and internal standard (IS, carbamazepine): 236.85 → 194.15 (Q1 Pre Bias: −24 V, CE: −20.0 V, Q3 Pre Bias: −18.0 V), respectively. The mass spectrometer parameters were as follows: nebulizer gas, 2.9 L/min; dry gas, 10.0 L/min; heating gas, 10.0 L/min; interface voltage, −4.5 kV; interface temperature, 300 °C; CID gas pressure, 230 kPa; DL temperature, 250 °C; and heat block temperature, 400 °C. The autosampler temperature was maintained at 4 °C.

Samples were prepared by adding 300 μL of cold acetonitrile and 10 μL of a solution containing 1 μg/mL internal standard into 100 μL of triptolide-containing cultures for protein precipitation. Then, the mixture was centrifuged at 12,000 rpm for 5 min. The supernatant was injected into the LC-MS/MS system for quantitative analysis of triptolide, with an injection volume of 1 μL.

### 4.4. Identification of the Metabolites of Triptolide by LC/MS^n^-IT-TOF

To identify the metabolites of triptolide, an LC/MS^n^-IT-TOF instrument equipped with an ESI ionization source was implemented. The analytes were separated using an Alltima C_18_ column (150 mm × 2.1 mm, 5 μm, Grace, England). The temperature of the column oven was 35 °C with a flow rate of 0.6 mL/min, and the split ratio was 6:4. The mobile phase consisted of formic acid and water (0.1: 100, *v*/*v*) (as mobile phase A) and acetonitrile (as mobile phase B). The binary gradient elution method (A:B) was as follows: 0.01 min, 60:40; 3.00 min, 60:40; 10.00 min, 30:70; 25.00 min, 5:95; 30.00 min, 5:95; 30.01 min, 60:40; and 33 min, stop. The mass spectrometry conditions were set as follows: CDL temperature, 160 °C; heating block temperature, 200 °C; nebulizing gas flow rate, 1.5 L/min; detector voltage, 1.76 kV; and collision energy, 70%. Automatic detection mode was used for fragmentation, with a primary *m/z* ratio ranging from 100 to 800 and a secondary *m/z* ratio ranging from 50 to 500.

### 4.5. In Vitro Incubation of Triptolide with Gut Microbiota

The colon contents of six SD rats were collected after sacrifice, and sterilized anaerobic medium (Beijing Solibao Technology Co., Ltd., Beijing, China) was added with an *m*/*v* ratio of 1:20 (g/mL), which was mixed evenly and purged with nitrogen after filtering. The mixture was preincubated at 37 °C for 60 min under anaerobic conditions. Ten microliters of triptolide in methanol (1 mg/mL) was added to a presterilized centrifuge tube, which was mixed with 900 μL of the preincubated mixture under anaerobic conditions. The drug was incubated with the gut microbiota 37 °C for 0, 6, 12, and 24 h. In addition, a negative control containing heat-inactivated gut microbiota was incubated with triptolide for the same amount of time (24 h). After incubation, a 3-fold volume of cold acetonitrile was added to the culture medium to stop the reaction and precipitate the protein. After centrifugation at 12,000 rpm for 5 min, the supernatants were removed and dried under nitrogen at room temperature. One hundred microliters of methanol:water (v:v = 1:1) was used for reconstitution, and 20 μL was injected for LC-MS/MS and LC/MS^n^-IT-TOF analysis.

### 4.6. In Vitro Incubation of Triptolide with Liver Microsomes

Fresh rat liver tissue was collected and homogenized with the addition of Tris/KCl (2 mL/g) to obtain microsomes. The resulting homogenate was centrifuged at 10,000 g for 25 min. Then, the homogenate was centrifuged at 105,000 g for 1 h to obtain microsomes in the pellet. The final microsome system contained 5 μL of microsomes, 2 μL of triptolide (0.2 mg/mL), 20 μL of NADPH, and 0.05 mM Tris/HCl (pH = 7.4), which made up a total volume of 200 μL. Then, the systems were incubated for 0, 15, 60, 90, and 120 min. After incubation, 300 μL of cold acetonitrile and 10 μL of a solution containing the internal standard (1 μg/mL) were added to 100 μL of the incubation solution for protein precipitation. The mixtures were centrifuged at 12,000 rpm for 5 min, and 1 μL of the supernatant was taken for quantitative analysis by LC-MS/MS. Another 3 volumes of cold acetonitrile were added to the remaining incubation solution. After centrifugation at 12,000 rpm for 5 min, the entire supernatant was dried under nitrogen at room temperature and reconstituted with 100 μL of methanol: water (*v*:*v* = 1:1) for the identification of metabolites.

### 4.7. Statistical Analysis

Data acquisition and processing were performed with Shimadzu LC-MS Solution (version 5.72, Kyoto, Japan). Two-tailed ANOVA and Student’s t-test were used for statistical analysis with GraphPad Prism Version 5 (GraphPad Software, San Diego, CA, USA). Data are expressed as the mean ± standard deviation (SD), and p values less than 0.05 were considered statistically significant.

## Figures and Tables

**Table 1:** Characteristics of triptolide metabolites in gut microbiota and liver microsomes by LC/MS^n^-IT-TOF.

	Metabolites	Reaction	Predicted Molecular Weight	Molecular Formula	Fragment Characteristics		
					MS^1^/[M + H]^+^	MS/MS	MS^3^
**Gut microbiota**	**M1**	**+2O, -2H**	**390**	C_20_H_22_O_8_	391	373, 355, 309	355, 337, 319, 309, 255
	M2	+2O, -2H	390	C_20_H_22_O_8_	391	373, 355, 309	355, 337, 319, 309, 255
	M3	-4H	356	C_20_H_20_O_6_	357	339, 321, 275, 247, 221, 161, 147	321
	M4	-4H	356	C_20_H_20_O_6_	357	339, 321, 275, 247, 221, 161, 147	321
**Liver microsomes**	M1	+2O, -2H	390	C_20_H_22_O_8_	391	373, 355, 309	355, 337, 319, 309, 255
	M2	+2O, -2H	390	C_20_H_22_O_8_	391	373, 355, 309	355, 337, 319, 309, 255
	M5	+O	376	C_20_H_25_O_7_	377	358, 302, 293, 219, 209	275, 218

## Supplementary Materials

Supplementary Materials are available online at https://www.mdpi.com/1420-3049/25/3/606/s1.

## References

[1] Bai S., Hu Z.Y., Yang Y., Yin Y.F., Li W.Y., Wu L.J., Fang M.R. (2016). Anti-Inflammatory and neuroprotective effects of triptolide via the NF-kappaB signaling pathway in a rat MCAO model. Anat. Rec. (Hoboken).

[2] Liu Q. (2011). Triptolide and its expanding multiple pharmacological functions. Int. Immunopharmacol..

[3] Ziaei S., Halaby R. (2016). Immunosuppressive, anti-inflammatory and anti-cancer properties of triptolide: A mini review. Avicenna J. Phytomed..

[4] Li X.J., Jiang Z.Z., Zhang L.Y. (2014). Triptolide: Progress on research in pharmacodynamics and toxicology. J. Ethnopharmacol..

[5] Zhao J., Xie C., Mu X.Y., Krausz K.W., Patel D.P., Shi X.W., Gao X.X., Wang Q., Gonzalez F.J. (2018). Metabolic alterations in triptolide-induced acute hepatotoxicity. Biomed. Chromatogr..

[6] Xu Y., Du J.B., Feng H.J., Zuo J.P., Xu H.T., Li Y.C., Zhong D.F. (2019). Studies on the metabolism of a triptolide derivative (5R)-5-hydroxytriptolide *in vitro*. Acta Pharm. Sin..

[7] Liu J., Li L., Zhou X., Chen X.Y., Huang H.H., Zhao S.B., Li X.L., Zhong D.F. (2013). Metabolite profiling and identification of triptolide in rats. J. Chromatogr. B.

[8] Liu J.Q., Wang X.M., Zhang G.H., Shu J.C., Zhang R. (2015). Studies on Triptolide Metabolites *in vivo*. J. Jiangxi Univ. Trad. Chin. Med..

[9] Obach R.S. (2013). Pharmacologically active drug metabolites: Impact on drug discovery and pharmacotherapy. Pharmacol. Rev..

[10] Sousa T., Yadav V., Zann V., Borde A., Abrahamsson B., Basit A.W. (2014). On the colonic bacterial metabolism of azo-bonded prodrugs of 5-aminosalicylic acid. Pharm. Sci..

[11] Haiser H.J., Gootenberg D.B., Chatman K., Sirasani G., Balskus E.P., Turnbaugh P.J. (2013). Predicting and manipulating cardiac drug inactivation by the human gut bacterium *Eggerthella lenta*. Science.

[12] Wallace B.D., Wang H., Lane K.T., Scott J.E., Orans J., Koo J.S., Venkatesh M., Jobin C., Yeh L.A., Mani S. (2010). Alleviating cancer drug toxicity by inhibiting a bacterial enzyme. Science.

[13] Zimmermann M., Zimmermann-Kogadeeva M., Wegmann R., Goodman A.L. (2019). Separating host and microbiome contributions to drug pharmacokinetics and toxicity. Science.

[14] Klatt N.R., Cheu R., Birse K., Zevin A.S., Perner M., Noël-Romas L., Grobler A., Westmacott G., Xie I.Y., Butler J. (2017). Vaginal bacteria modify HIV tenofovir microbicide efficacy in African women. Science.

[15] Peng Z.H., Wang J.J., Du P., Chen Y. (2012). Identification of *in vivo* and *in vitro* metabolites of triptolide by liquid chromatography–tandem mass spectrometry. J. Pharm. Biomed. Anal..

[16] Sun S.T., Jin Y., Yuan B., Jiang X., Xu H.Y. (2013). Analysis of metabolites of triptolide and triptolide in rats. Chin. J. Pharm. Ind..

[17] Shu D.F., Li R.L., Sun Y.J. (1990). Comparison of triptolide with the ethyl acetate extract of Tripterygium wilfordii in the treatment of rheumatoid arthritis. Chin. Trad. Herb. Drugs.

[18] Zheng Y.L., Lin J.F., Lin C.C., Xu Y. (1994). Anti-inflammatory effect of triptolide. Acta Pharmacol. Sin..

[19] Li W., Liu Y., He Y.Q., Zhang J.W., Gao Y., Ge G.B., Liu H.X., Huo H., Liu H.T., Wang L.M. (2008). Characterization of triptolide hydroxylation by cytochrome P450 in human and rat liver microsomes. Xenobiotica.

