# Supplementary material for: Transforming of Triptolide into Characteristic Metabolites by the Gut Microbiota
Source: Molecules. 2020 Jan 30;25(3):606. doi: 10.3390/molecules25030606 (PMC7037371; doi:10.3390/molecules25030606)
Supplement: Supplementary file 1 [file molecules-25-00606-s001.pdf]

Supplementary Materials

Validation of triptolide in gut microbiota by LC-MS/MS

Calibration standards and QC preparation

The incubation culture of gut microbiota without triptolide (90 μL) was mixed with 10 μL of working solutions of the calibration standards and 10 μL of carbamazepine (1 μg/mL), then precipitated with 300 μL of acetonitrile. After vortexing, samples were centrifuged at 12,000 rpm for 5 min. The standard curve concentrations of triptolide were as follows: 0.5, 1, 2, 10, 20, and 100 μg/mL. The concentrations of QCs were 0.5 μg/mL (for low concentration of quality control, LQC), 10 μg/mL (median concentration of quality control, MQC), and 100 μg/mL (high concentration of quality control, HQC).

Method validation

The method was validated in terms of specificity, linearity, intra- and inter-day accuracy and precisions, recovery, matrix effect and stability according to the validation guidelines of biological sample analysis by Chinese State Food and Drug Administration.

Specificity

The specificity of the method was assessed by analyzing the blank incubation culture of gut microbiota and the culture spiked with triptolide, or IS, respectively.

Linearity

Linearity was determined by plotting the peak area ratio of analytes to internal standard against the theoretical concentrations of the standards with weighted (1/*c*) least square linear regression.

Precision and Accuracy

The inter- and intra-day precision and accuracy were carried out by analyzing repeated quality control samples (LQCs, MQCs, HQCs, n = 5) on three consecutive days. The precision and accuracy was expressed as the relative standard deviation (RSD, %) and RE (%), respectively.

Recovery and Matrix Effect

Recovery was calculated by comparing the peak area of five replicates of QC samples (LQCs, MQCs, and HQCs) with the peak area of the post-treatment spiked samples. The matrix effects were calculated by comparing the peak area of the QCs samples to that of the non-matrix samples with identical level of standard solutions.

Stability

The stability of the analytes in gut microbiota was investigated by analyzing the pre-treated QC samples stored at room temperature for 4h and the post-treated QC samples placed in the autosampler at 4℃ for 24 h.

Results

The mass spectra of triptolide and the internal standard (carbamazepine) in gut microbiota of rats are shown in Figure 1(B) and the retention times of triptolide and the internal standard were 4.2 min and 4.7 min, respectively.

The calibration curves of the analytes were linear, with correlation coefficients $r^2 > 0.99$, and the linear range of triptolide was 0.5-100 μg/mL.

Accuracy and precision results showed that the measured values were within the ±15% deviation and met the requirements.

The recovery and matrix effect results were in the range of 83.89-105.20% and 83.57-92.12%, respectively, suggesting that the sample processing method was proper for the extraction of triptolide and the matrix effect was weak.

Results of stability showed that triptolide was stable under the analytical process of samples.

These data demonstrated that the developed method was reliable and reproducible for the quantitative analysis of triptolide in gut micobiota.

Table S1 Validation results of triptolide in gut microbiota by LC-MS/MS (*n* = 5)

	LQC	MQC	HQC
**Precision and Accuracy**			
**Intra-day**			
**Batch 1**			
Mean (μg/mL)	0.4962	10.90	97.98
RE (%)	-0.76	8.95	-2.02
RSD (%)	5.89	2.00	4.52
**Batch 2**			
Mean (μg/mL)	0.4958	10.86	102.36
RE (%)	-0.84	8.59	2.36
RSD (%)	4.08	1.41	3.25
**Batch 3**			
Mean (μg/mL)	0.5208	11.11	98.01
RE (%)	4.16	11.14	-1.99
RSD (%)	4.31	2.68	3.94
**Inter-day**			
Mean (μg/mL)	0.5043	10.96	99.45
RE (%)	0.85	9.57	-0.45
RSD (%)	5.36	2.38	4.44
**Recovery**			
Mean (%)	105.20	96.38	83.89
RSD (%)	8.79	9.04	2.01
**Matrix Effect**			
Mean (%)	92.12	91.27	83.57
RSD (%)	10.88	7.39	2.25
**Stability**			
**At Room Temperature for 4 h Before Treatment**			
Mean (%)	90.64	116.33	92.43
RSD(%)	4.65	2.95	6.72
**In the Autosampler for 24h at 4℃ Post-Treatment**			
Mean (%)	94.76	111.46	87.20
RSD(%)	8.67	3.84	4.88

Table S2 The peak area ratio of the metabolites versus triptolide in gut microbiota

Ratio (%)	0 h	6 h	12 h	24 h
M1	1.34±0.07	2.85±0.14	4.04±0.47	5.78±0.77
M2	1.98±0.32	2.28±0.11	3.61±0.21	5.63±0.13
M3	0.35±0.03	0.95±0.15	1.61±0.15	5.24±0.24
M4	2.50±0.65	4.12±0.42	7.58±0.13	7.93±0.35
